# Store-Operated Ca^2+^ Entry in Tumor Progression: From Molecular Mechanisms to Clinical Implications

**DOI:** 10.3390/cancers11070899

**Published:** 2019-06-27

**Authors:** Yih-Fung Chen, Peng-Chan Lin, Yu-Min Yeh, Li-Hsien Chen, Meng-Ru Shen

**Affiliations:** 1 Graduate Institute of Natural Products, College of Pharmacy, Kaohsiung Medical University, Kaohsiung 80708, Taiwan; 2 Department of Medical Research, Kaohsiung Medical University Hospital, Kaohsiung 80708, Taiwan; 3 Department of Internal Medicine, National Cheng Kung University Hospital, College of Medicine, National Cheng Kung University, Tainan 70101, Taiwan; 4 Department of Pharmacology, College of Medicine, National Cheng Kung University, Tainan 70101, Taiwan; 5 Department of Obstetrics and Gynecology, National Cheng Kung University Hospital, College of Medicine, National Cheng Kung University, Tainan 70101, Taiwan

**Keywords:** Ca^2+^ signaling, orai, stromal interaction molecule (STIM), store-operated Ca^2+^ entry (SOCE), migration

## Abstract

The remodeling of Ca^2+^ homeostasis has been implicated as a critical event in driving malignant phenotypes, such as tumor cell proliferation, motility, and metastasis. Store-operated Ca^2+^ entry (SOCE) that is elicited by the depletion of the endoplasmic reticulum (ER) Ca^2+^ stores constitutes the major Ca^2+^ influx pathways in most nonexcitable cells. Functional coupling between the plasma membrane Orai channels and ER Ca^2+^-sensing STIM proteins regulates SOCE activation. Previous studies in the human breast, cervical, and other cancer types have shown the functional significance of STIM/Orai-dependent Ca^2+^ signals in cancer development and progression. This article reviews the information on the regulatory mechanisms of STIM- and Orai-dependent SOCE pathways in the malignant characteristics of cancer, such as proliferation, resistance, migration, invasion, and metastasis. The recent investigations focusing on the emerging importance of SOCE in the cells of the tumor microenvironment, such as tumor angiogenesis and antitumor immunity, are also reviewed. The clinical implications as cancer therapeutics are discussed.

## 1. Introduction

The important cellular activities, including survival, death, proliferation, motility, and contraction, depend on intracellular Ca^2+^ signaling, which mainly acts as an intracellular second messenger, transmitting extracellular stimulation into specific intracellular signaling cascades. The abnormal regulation of intracellular Ca^2+^ homeostasis has been associated with human diseases, including developmental defects, immune disorders, cardiovascular diseases, neurodegenerative diseases, and tumorigenesis [1]. The growing evidence indicates the dysregulated Ca^2+^ signals as an important contributor to many hallmark properties of cancer [2,3,4]. Among the Ca^2+^ entry mechanisms, store-operated Ca^2+^ entry (SOCE) mediated by Orai and stromal interaction molecule (STIM) is a relatively recently identified one [5]. SOCE shows the impact on a variety of physiological and pathological mechanisms, especially in immune systems [6,7,8], and has been implicated with the pathogenesis of different cancer types [9,10,11]. Increasing evidence clarifying the biological functions of STIM and Orai proteins as well as their roles in cancer cells have made them potential prognostic biomarkers or cancer therapeutic targets. Here we update an overview of recent advances on the crucial roles and the molecular mechanisms of SOCE pathways in cancer biology, particularly proliferation, cell death, migration, invasion, resistance, as well as the interactions between components of the tumor microenvironment. Moreover, the clinical implications of STIM and Orai proteins and the emerging development of SOCE mechanisms as the selective target for cancer therapeutics are discussed.

## 2. Regulation of Ca^2+^ Homeostasis by Store-Operated Ca^2+^ Entry (SOCE)

Cells can induce intracellular Ca^2+^ signals through the entry of extracellular Ca^2+^ or the release of organellar Ca^2+^ from ER or mitochondria. SOCE that is constituted of the tight coupling of these two pathways can be activated through the following steps [12], including (i) the generation of inositol-1,4,5-trisphosphate (IP3) via the effects of phospholipase C (PLC) upon the activation of cell surface receptor, (ii) loss of ER Ca^2+^ into cytosol through the gating of ER Ca^2+^ release channel IP3R by IP3, and (iii) influx of extracellular Ca^2+^ through store-operated Ca^2+^ (SOC) channels in response to ER Ca^2+^ depletion. Such Ca^2+^ influx provides Ca^2+^ not only for the refilling of the ER store but also for the transduction of Ca^2+^ signaling pathways.

### Molecular Basis of SOCE: The Dynamic Interaction between ER Ca^2+^ Sensors STIM Proteins and Cell Surface SOC Channel Orai Molecules

The molecular identities underlying SOCE activation are [6,13]: the SOC channel pore-forming Orai proteins, Orai1-Orai3, and the ER Ca^2+^-sensing STIM molecules STIM1 and STIM2, respectively. Compared with the extensive research focus on STIM1 and Orai1, lesser is known about the other isoforms STIM2, Orai2, and Orai3 [14].

Mammals express three types of Orai proteins [15,16], of which Orai1 is the most extensively characterized and Orai3 is a unique one as whose expression is only found in mammals [15]. All three Orai proteins are reported to complex with STIM1 to form functional SOC channels, but their tissue distribution and the selectivity and conductivity for Ca^2+^ are different [15]. Orai2 and Orai3 can be activated by store depletion, albeit with different efficacies, but the SOC currents induced by Orai2 and Orai3 are smaller than those induced by Orai1 [14].

STIM molecules are single-pass transmembrane proteins mainly localized in the ER [13]. Some relevant functional domains in the luminal N-terminus of STIM molecules include an EF-hand motif for ER Ca^2+^ store sensing and a sterile α motif for STIM oligomerization. The cytosolic C-terminal domains of STIM mediate protein–protein interactions and SOC channel activation. Although STIM1 and STIM2 have a high degree of structural homology, they show the critical differences which impact the functions and properties. For example, STIM1 is less sensitive to minor alterations in ER Ca^2+^ due to the higher Ca^2+^ affinity of its EF-hand motif [17]. In contrast, due to the lower Ca^2+^ affinity of STIM2, STIM2 can be activated by the minor changes in ER Ca^2+^ [17]. Moreover, STIM1 activates SOCE more efficiently, potentially due to the faster aggregation kinetics and a stronger Orai targeting efficacy [18,19]. Hence, STIM1, as the primary sensor of ER Ca^2+^, promotes SOCE upon strong stimuli that induce a rapid and substantial storage depletion [20,21], whereas STIM2, as a housekeeping ER Ca^2+^ sensor, stabilizes ER Ca^2+^ levels after the slower and weak-to-moderate store depletion [21,22].

The STIM molecules play a dual role as the ER Ca^2+^ sensor and SOCE activator. Loss of ER luminal Ca^2+^ leads to the aggregation of STIM into multiple puncta and promotes the binding of activated STIM proteins to microtubule plus-end-binding protein EB1 [23]. Such interaction is important for STIM1 trafficking towards the ER-plasma membrane junctions [24,25], where they bind to and turn on Orai proteins to allow Ca^2+^ entry. This mechanism was challenged by a recent study on HeLa cells showing that EB1 binding may delay STIM1 translocation during ER Ca^2+^ loss, prevent overloading of ER Ca^2+^, and avoid excessive activation of SOCE [26]. However, a very recent study on cervical cancer SiHa cells showed that STIM1 and STIM2 displayed different kinetic characteristics during SOCE activation [27]. Results from high-resolution imaging and total internal reflection fluorescence microscopy of living cells demonstrated that a decrease in ER Ca^2+^ levels promoted the oligomerization, EB1 association and membrane translocation of STIM1. In contrast, STIM2 was constitutively aggregated, without prominent trafficking or microtubule association even under ER Ca^2+^ depletion. Further work needs to clarify if the microtubule-dependent SOCE activation is context-dependent, perhaps in tumor cells expressing constitutively-activated STIM2.

## 3. Diagnostic and Prognostic Values of STIM/Orai in Human Cancers

The most extensively examined molecules of SOCE in tumor biology are STIM1 and Orai1. STIM1 was initially identified as a tumor suppressor gene in human rhabdoid tumor and rhabdomyosarcoma cell lines [28]. However, later studies revealed the opposite functions of STIM1 in promoting a diversity of malignant characteristics, including proliferation or resistance to apoptosis, migration, invasion, and metastasis [9,10,11].

Aberrant overexpression of STIM1 or Orai1 and thus upregulated SOCE activity have been observed in several types of human cancers. For instance, STIM1 or Orai1 is overexpressed in tumor tissues when compared with noncancerous or precancerous tissues in patients with breast [29], cervical [24,27,30,31], colorectal [32,33], liver [34], lung [35], clear cell renal cancers [36], or multiple myeloma [37]. Furthermore, the expression of Orai1/STIM1, as well as SOCE activity, is enhanced in cisplatin-resistant ovarian carcinoma cells when compared with the therapy-sensitive parental cells [38]. In contrast, in prostate cancer, which could be the only exception, Orai1 expression was decreased when compared to normal tissue but was increased when compared to hyperplasia [39].

Tumor expression of Orai1 and STIM1 have substantial implications for the adverse prognosis of cancer patients. The studies in human cervical cancer indicated that STIM1 upregulation in primary tumors was significantly correlated with the poorer clinical outcomes, such as larger tumor size and elevated lymph node metastasis [30]. Overexpression of STIM1/Orai1 in multiple myeloma patients was closely associated with the shorter progression-free survival [37]. In patients with esophageal squamous cell carcinoma, the poorer overall, as well as recurrence-free survival, was characterized by high expression of Orai1 in tumor tissue [40]. These highlight the clinical significance of STIM1 and Orai1 in tumor progression.

Interestingly, the specific distribution of overexpressed STIM1 in the invasive tumor front was identified in a recent study on human cervical cancer [41]. Results from the simultaneous immunostaining of STIM1 and STIM2 showed that, despite the overexpression of both isoforms in tumor tissues, STIM1 is the principle ER Ca^2+^-sensing molecule detected in the invasive tumor front [27]. These imply that STIM1 is associated with both tumor growth and invasion, whereas STIM2 is mainly correlated with tumor growth. Further studies would contribute to the understandings of the clinical implications of such patterns of STIM1/STIM2 expression and the grades of STIM1 distribution in the invasive tumor front.

The clinical significance of STIM2 is relatively more complicated when compared with STIM1. Increased STIM2 expression was also found in glioblastoma multiforme tumors [42]. STIM2 was overexpressed in 64% of human colon cancers examined, but increased expression of STIM2 was significantly correlated with a less invasive phenotype, suggesting a tumor growth suppressing role of STIM2 [43]. High expression levels of STIM2 was found in tumor stroma and epithelial tissues of human prostate cancer [44]. However, downregulation of STIM2 in the stroma region was correlated with the transition from moderate-to-high Gleason grade, which is often used as a prognostic marker for prostate cancer [44]. A microarray study of breast tumor revealed that the high STIM1/STIM2 expression profiles accompanied by augmented SOCE were correlated with the breast cancer subtype with the poorest prognosis, suggesting the clinical significance of STIM1/STIM2 ratio in breast cancer [45]. Although most studies indicated a potential tumor suppressive action of STIM2, a recent study on a limited number of surgical specimens of cervical cancer showed a decreased tumoral STIM2 expression when compared with noncancerous epithelium, but a higher tumoral STIM2 level when compared with invasive tumor front [27]. In human melanoma, STIM2 and Orai1 were highly expressed in primary human melanomas and elevated in the invasive rim of the lymph node metastatic tumors [46]. Therefore, the use of the STIM1/STIM2 ratio as a marker of tumor aggressiveness might be promising and worth further evaluated.

The clinical significance of Orai3 has just emerged from studies on lung cancer [47,48]. A small cohort study on human lung adenocarcinoma (*n* = 60) reported that the immunostaining of Orai3 was elevated in lung cancer tissues as compared to the matched nontumorous ones, and, moreover, correlated with a high tumor grade [47]. Another large cohort of lung adenocarcinoma samples (*n* = 200) conducted by the same research group further demonstrated the association of the Orai3 immunostaining with the aggressiveness of lung adenocarcinoma [48]. These studies suggest the potential of Orai3 overexpression as an independent prognostic marker for the early-stage lung adenocarcinoma.

The main studies demonstrating the diagnostic and prognostic values of STIM and Orai proteins in human cancers are summarized in Table 1.

## 4. Importance of SOCE Signals in Key Hallmarks of Cancer Cells

It is well-accepted that during the multistep tumor development cancer cells acquire a variety of malignant characteristics, such as proliferation, migration, invasion, and metastasis [2,3]. Growing studies demonstrated the STIM/Orai-mediated SOCE function as dynamic coordinators of intracellular Ca^2+^ signals that regulate the variety of cancer-associated processes and pathways [9,13,49]. Below, we discuss the up-to-date recent studies on the specific contributions of STIM and Orai isoforms to the selective regulation of oncogenic and tumor suppressor pathways.

### 4.1. Proliferation and Cell Cycle Regulation

The functional importance of STIM1/Orai1-mediated SOCE in cancer cell proliferation was extensively studied. A recent study demonstrated that SOCE mediated STIM1 and Orai1 is the molecular basis for Ca^2+^ microdomain controlling the G1/S checkpoint of the cell cycle [31]. The SOCE activity fluctuated during cell cycle progression in different cell types. Mechanistic studies in cervical cancer cells showed that inhibition of SOCE by pharmacological blockers or silencing of STIM1 or Orai1 reduced the phosphorylation of the cyclin-dependent kinase CDK2 and upregulated cyclin E expressions, resulting in the cell cycle arrest in G1/S transition accompanied with autophagy [31]. Furthermore, STIM1 knockdown significantly inhibited cell proliferation of human cervical cancer cells by slowing down the cell cycle progression accompanied by increasing cyclin-dependent kinase inhibitor p21 protein and decreasing phosphatase Cdc25C protein levels [30]. Similar phenomena were found in another type of cancer cells, such as glioblastoma cell [50]. STIM1 silencing slowed cell proliferation by arresting cell cycle at G0/G1 phase in glioblastoma cell lines, attributed to the regulation of the p21, cyclin D1, and CDK4. The pro-proliferative role of STIM1 in vivo was further demonstrated by STIM1-knockdowned xenografts of human glioblastoma or cervical cancer, which exhibited an attenuated growth rate as compared to control tumors [30,50]. These studies highlight the important roles of STIM1/Orai1-mediated SOCE pathway in the regulation of the cell cycle checkpoint and thereby controlling cell proliferation.

As for Orai3, although less studied, most current reports supported its pro-proliferative and pro-tumorigenic roles. It has been demonstrated that SOCE in estrogen receptor (ER)-positive breast cancer cells is mediated by Orai3 and STIM2/STIM1, whereas SOCE in ER-negative breast cancer cells mostly depends on the canonical Orai1/STIM1 pathway [51]. Orai3 silencing reduced the in vitro anchorage-independent growth and in vivo tumor xenograft growth of ER-positive MCF-7 breast cancer cells [52]. RNAi-mediated inhibition of Orai3 in MCF-7 cells arrested cell cycle progression at the G1 phase through downregulating the proto-oncogene c-myc pathway and accumulating tumor-suppressor p53 and cyclin-dependent kinase inhibitor p21, accompanied with a marked decrease in CDK4, and CDK2 and pERK1/2 [53,54]. These results demonstrate the potentials of Orai3 as a selective therapeutic target for tumor growth of ER-positive breast cancers. The association of Orai3 expression with the proliferation of prostate cancer LNCaP cells has also been reported [55]. The enhanced Orai3 expression favors the heteromerization with Orai1, which supports the store-independent Ca^2+^ entry, thereby promoting cell proliferation and decreasing apoptosis via NFAT signaling and the cell cycle protein cyclin D1 [55]. Regarding the role of STIM2 in cell cycle regulation, results from the individual or simultaneous silencing of STIM1/STIM2 in cervical cancer cells suggested that both STIM1 and STIM2 are involved in the regulation of SOCE during G1/S transition [31], and thus contribute to cell proliferation [27].

### 4.2. Cell Death: Apoptosis and Autophagy

The intrinsic ability to evade apoptosis is a major hallmark of cancer that also causes tumor development and resistance to treatment. Many studies demonstrated the importance of SOCE in the apoptosis of cancer cells, but the specific roles of STIM1 and Orai molecules are controversial. The essential role of the functional coupling of STIM1 to Orai1, as well as STIM1/Orai1-dependent SOCE, in promoting apoptosis was firstly demonstrated in the study on prostate cancer cells [56]. Orai1 knockdown or ectopic expression of Orai1 dominant-negative mutants in prostate cancer cells downregulated SOCE activity and decreased susceptibility to diverse apoptosis-inducing stimuli including thapsigargin, tumor necrosis factor-α (TNF-α) and cisplatin. Moreover, the expression of exogenous Orai1 in the androgen-independent prostate cancer cells, which express a low level of Orai1 accompanied with attenuated SOCE, was found to restore the normal rate of apoptosis of these cells [56]. These suggest that endogenous SOCE mediated by Orai1 has a pivotal role in triggering apoptosis of prostate cancer cells.

The potential link of STIM1 to promoting apoptosis came from a recent investigation into the functional role of microRNA-185 in chemotherapeutic sensitivity of gastric cancer [57]. STIM1 is a direct target of miR-185 in colorectal cancer cells, in which miR-185 inversely correlates with the expression of STIM1 and associated with the progression of colorectal cancer [33]. Ectopic expression of miR-185 in gastric cancer cells increased the sensitivity to low-dose chemotherapeutic drugs, which did not provoke significant apoptosis themselves [57]. In contrast, high-dose chemotherapy-induced apoptosis of gastric cancer cells was attenuated by the silencing of endogenous miR-185 [57]. However, neither STIM1 expressions nor SOCE activity was examined in these cells.

In contrast to the above mentioned proapoptotic roles for STIM1 and Orai1, the apoptotic resistant roles of STIM1 and Orai1 proteins were found in other types of cancer. STIM1 or Orai1 silencing in the pancreatic adenocarcinoma Panc1 cells lead to increased apoptosis induced by gemcitabine and 5-fluorouracil [58]—the two common chemotherapy drugs. Similarly, in multiple myeloma cells, inhibition of SOCE or silencing of STIM1 or Orai1 reduced cell viability and caused cell cycle arrest and apoptosis [37]. Interestingly, the antiapoptotic effects of Orai1 were also reported in breast cancer MCF-7 and T47D cells cultured in collagen-coated conditions [59,60]. Although the SOCE in these ER-positive breast cancer cells is mainly mediated by Orai3 [51], collagen can promote Orai1 expressions and surface localizations, basal Ca^2+^ entry through Orai1, and ERK1/2 phosphorylations, and thereby promote the antiapoptotic effects. Moreover, Orai1 silencing can reverse the antiapoptotic effects of collagen in breast cancer MCF-7 and T47D cells.

A role of STIM2 in promoting apoptosis resistance has been reported in colorectal cancer carcinoma cells [61]. The expression of STIM2 in human colon cancer HT29 cells was significantly lower than that in normal mucosa NCM460 cells. Interestingly, reduced ER Ca^2+^ levels, abolished SOCE, and lower hydrogen peroxide-induced apoptosis was found in normal mucosal cells with STIM2 silencing. These data suggest that downregulation of STIM2 may underlie the apoptosis resistance in colon cancer cells.

Autophagy is a physiological process by which cellular material is delivered to lysosomes for degradation and recycling and thus aid cell survival during starvation [62]. The role of autophagy in cancer is dynamic and depends, in part, on tumor stage [63,64]. It is thought that autophagic cell death prevents initial cancer development. Conversely, once the cancer is established, increased autophagy enhances survival, growth, and metastasis of tumor cells subjected to metabolic or other environmental stresses. Emerging studies demonstrated the importance of STIM1/Orai1-mediated SOCE in the context-dependent roles of autophagy in human cancers. A recent study reported that resveratrol induced autophagic cell death of human prostate cancer cells through regulating SOCE pathways, including downregulating STIM1 expression and decreasing ER Ca^2+^ stores [65]. The reduced ER Ca^2+^ levels triggered ER stress and inhibited AKT/mTOR signaling pathways, which eventually resulted in autophagic cell death [65]. Pharmacological or siRNA inhibition of the STIM1/Orai1 pathway of SOCE activation in human cervical cancer cells resulted in the decreased phosphorylation of CDK2 and the excursive expression of cyclin E, leading to autophagy accompanied with a cell cycle arrest in G1/S transition [31]. A recent study in human hepatoma cells showed that 5-fluorouracil induced autophagy by decreasing Orai1-dependent SOCE activation [66]. Both Orai1-specific silencing and non-specific SOCE inhibition with SKF-96365 augmented the 5-fluorouracil-induced autophagic cell death [66]. In contrast, SOCE inhibition by SKF-96365 in human colorectal cancer cells induced cell cycle arrest and apoptosis with concomitant cytoprotective autophagy accompanied with the inhibition of calcium/calmodulin-dependent protein kinase IIγ (CaMKIIγ)/AKT signaling cascade [67]. These studies highlight the unique roles for the STIM1/Orai1-mediated SOCE in regulating autophagy. The opposing roles of SOCE in autophagic cell death or cytoprotective autophagy may be stress- or cell-type dependent.

### 4.3. Resistance and Stemness

Intrinsic or acquired drug resistance is one of the significant challenges for the effective treatment of cancer. Studies assessing how SOCE-dependent Ca^2+^ signals regulate the response to therapeutic agents and the acquisition of therapeutic resistance in cancer cells are emerging. A study using paired ovarian carcinoma cell lines with different cisplatin sensitivities showed that SOCE participates in therapy resistance through elevated Orai1/STIM1 expressions and SOCE activity, accompanied by increased AKT activity [38]. Similarly, upregulated STIM1 expression was found in chemo-resistant osteosarcoma tissues and cisplatin-resistant osteosarcoma cells [68]. Mechanistically, STIM1, as well as Ca^2+^ entry, contributes to cisplatin resistance of osteosarcoma cells by inhibiting ER stress-mediated apoptosis. Moreover, patients with positive STIM1 expression exhibited a poorer overall survival than those with negative STIM1 expression. A recent study in gastrointestinal stromal tumors showed that STIM1-dependent SOCE activation contributes to resistance against imatinib, a small molecule kinase inhibitor, though the MEK/ERK pathway [69]. A vital role for STIM1/Orai1-mediated SOCE in resistance against rituximab, the anti-CD20 monoclonal antibody used for treating B-cell malignancies, has also been demonstrated [70]. Rituximab stimulates STIM1-Orai1 clustering and activates SOCE in human B lymphoma cell lines. Inhibition of SOCE with pharmacological inhibitors or Orai1 silencing in human B lymphoma cell lines resulted in higher apoptosis and enhanced caspase activation upon rituximab treatment. Results from the mouse xenograft lymphoma model showed that Orai1 knockdown promoted the therapeutic efficacy of rituximab.

The importance of Orai3 in conferring chemotherapy resistance has been highlighted in a recent study employing large human breast cancer data sets [71]. Overexpression of Orai3 reduced the sensitivity of cell death response to chemotherapeutic agents, including cisplatin, 5-fluorouracil, and paclitaxel in ER-positive T47D breast cancer cells. Mechanistic investigations revealed that Orai3 conferred resistance by downregulating the tumor suppressor protein p53 via the prosurvival PI3K/Sgk-1 signaling pathway in a Ca^2+^-dependent manner.

Cancer stem-like cells (CSCs), which are small subpopulations of tumor cells with self-renewal capacity, are one of the critical factors contributing to the establishment, progression, metastasis, and recurrence of tumors, also potentially responsible for drug resistance [72,73]. A study on oral/oropharyngeal squamous cell carcinoma provided emerging clues of an association between SOCE and the stemness properties of cancer cell [74]. Orai1 expression was elevated during oral/oropharyngeal carcinogenesis and highly expressed in CSC-enriched populations of human oral/oropharyngeal squamous cell carcinoma. Ectopic Orai1 expression enhanced the conversion of non-tumorigenic immortalized oral epithelial cells into malignant cells with CSC properties, such as self-renewal capacity, increased stemness transcription factors, and enhanced mobility, through upregulating Ca^2+^ dependent NFATc3 signaling.

Collectively, these studies suggested that SOCE-dependent Ca^2+^ signal is a potential strategy to overcome cancer stemness and improve the efficacy of chemotherapeutic agents for cancer treatment.

### 4.4. Cancer Cell Motility

Tumor metastasis, the dissemination of the primary tumor to distant organs, requires the migration and invasion of cancer cells [3]. This multistep spreading process involves the proteolysis of the extracellular matrix (ECM), migration and local invasion of cancer cells into neighboring connective tissues, intravasation into bloodstreams and lymphatic vasculature, and extravasation and colonization into distant organs, finally resulting in the formation of secondary metastatic lesions [75]. Intracellular Ca^2+^ signaling plays a vital role in cell migration of both malignant and nonmalignant cells through orchestrating cytoskeletal reorganization, focal adhesions, and front–rear end polarity [76,77]. In migratory cells, the membrane extensions with directed actin polymerization and nascent focal adhesions are often found at the leading edge, whereas the disassembled cell adhesions and actin cytoskeleton with membrane retractions mediated by myosin II contraction are found at the rear end [25,78]. The contraction of myosin II-based actomyosin is principally modulated by the phosphorylation of myosin II regulatory light chain by the Ca^2+^-dependent myosin light chain kinase (MLCK) [79,80]. The degradation of focal adhesion proteins, such as focal adhesion kinases, integrins, vinculin, and talin, by the Ca^2+^-sensitive protease calpain, underlies the disintegration of cell adhesions [81].

The critical roles of STIM1 and Orai1 proteins in tumor cell migration and the underlying molecular mechanisms have been extensively studied. The canonical STIM1/Orai1-mediated SOCE mechanism has been found to be required for cell migration of several cancer types, including breast cancer [82], cervical cancer [30], gastric cancer [83], colorectal cancer [32,33], hepatocellular carcinoma [34], renal cell carcinoma [36], nasopharyngeal carcinoma [84], glioma [85], and melanoma [86]. The role of STIM1 and Orai1 in cancer migration and metastasis initially came from studies in breast cancer and cervical cancer [30,82]. In breast cancer MDA-MB-231 cells, upregulation of Orai1 or STIM1 significantly increased the in vitro migratory capability and tumor metastasis potential in mice, whereas silencing of Orai1 or STIM1, as well as SOCE blockade with SKF-96365, attenuated these effects [82]. Studies in cervical cancer showed that STIM1-dependent SOCE is crucial for the migratory capability of cancer cells, as demonstrated by results from overexpression or silencing of STIM1 as well as the blockade of SOCE by 2-APB and SKF-96365 [30,87].

The molecular mechanisms by which STIM1/Orai1-dependent SOCE regulates tumor cell migration were highlighted in studies on breast cancer and cervical cancer [30,82,87]. Results from siRNA-mediated silencing revealed that STIM1 and Orai1-mediated SOCE affects focal adhesion turnovers—a crucial step in cell migration [82]. The defects in focal adhesion turnover and cell migration in breast cancer cells with STIM1/Orai1 silencing or SOCE blockade were rescued by the small GTPases Ras and Rac [82]. Studies in cervical cancer cells demonstrated that STIM1 regulates EGF-stimulated migration through the activation of several Ca^2+^-dependent molecules such as cytosolic tyrosine kinase Pyk2, calpain protease, and MLCK, as well as the recruitment of the active focal adhesion proteins, including Pyk2, FAK, and talin, and thereby facilitating focal adhesion turnovers, cytoskeleton reorganization, and actomyosin-mediated mechanotransduction [30,87]. The similar roles of STIM1 in focal adhesion turnover and cell migration were also found in hepatocellular carcinoma cells [34].

A pro-migratory role of STIM2/Orai1-mediated SOCE has also been reported in melanoma [46]. Interestingly, melanoma cells with Orai1 or STIM2 silencing exhibited the reduced migratory and invasive capabilities accompanied with upregulated cell proliferation, indicating that the switch from a pro-proliferative to a pro-migratory phenotype of melanoma is dependent on the SOCE mediated by Orai1 and STIM2 [46]. It was also reported that Orai1/Orai2-mediate SOCE regulates cell migration and FAK phosphorylation of human leukemia HL60 cells [88].

Invadopodia, also called as podosomes in nonmalignant cells, are dynamic actin-enriched cell protrusions that facilitate cancer cell invasion by ECM degradation [89]. To assemble the core of invadopodia structure requires the activation of the non-receptor tyrosine kinase Src and the localized production of phosphatidylinositol-3,4-bisphosphate [90,91,92]. During the maturation phase, invadopodia become proteolytically active through the recruitment of membrane type 1 (MT1)-matrix metalloproteinase (MMP) and local secretion of MMP2 and MMP9, and thereby mediating localized degradation of ECM and promoting cell invasion [93]. Recent studies on melanoma, breast cancer cells and v-Src-transformed mouse embryonic fibroblasts (MEFs) uncover STIM1 dependent SOCE as the principle Ca^2+^ signals that control invadopodia formation [41,94]. Blockade of SOCE activation with specific silencing of Orai1 or STIM1 silencing or phrenological inhibitors of SOCE affected invadopodia formation and reduced cell invasiveness in these cells. Mechanistically, STIM1/Orai1-mediated Ca^2+^ oscillations promoted the assembly of invadopodia precursors in melanoma cells by activating Src kinase [94]. The inhibition of SOCE abrogated the recycling of MMP-containing vesicles to the plasma membrane and resulted in an inhibited ECM degradation [94]. Live cell imaging in v-Src-transformed MEFs showed that STIM1 silencing significantly altered the invadopodia dynamics, shortened the maintenance phase of invadopodia, and reduced matrix degradation [41]. Moreover, the distribution of actomyosin around invadopodia was altered by STIM1 silencing, suggesting the maintenance of invadopodia structure through STIM1-mediated mechanotransduction [41]. These studies unravel a novel mechanism by which SOCE-mediated Ca^2+^ influx regulates the formation and maintenance of the invadopodia structures and the activity of proteolytic enzymes like MMPs, and thereby support cancer cell invasion.

**Figure 1:**
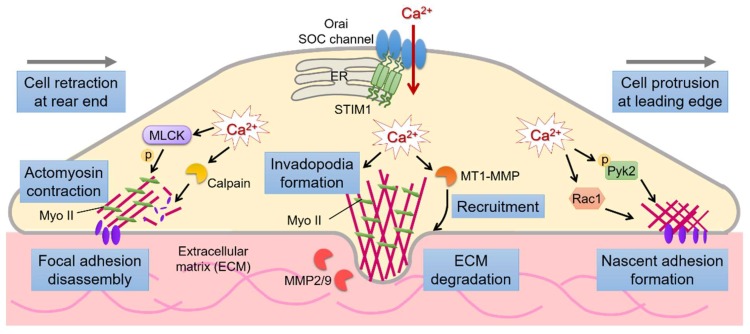
STIM1-mediated Ca^2+^ influx regulate the invasive migration of cancer cells through focal adhesion turnover, actomyosin contractility, and invadopodia formation. STIM1-dependent Ca^2+^ signaling integrates the dynamic interactions between actomyosin contraction and focal adhesion turnover to mediate efficient cell migration. STIM1-dependent Ca^2+^ signals also promote cancer cell invasion through the formation and maintenance of the invadopodia structures and the activation and incorporation of proteolytic enzymes like MMPs and MT1-MMP.

The mechanisms by which STIM1-mediated Ca^2+^ signals regulate the invasive migration of cancer cells through focal adhesion turnover, actomyosin contractility, and invadopodia formation are summarized in Figure 1.

Mitochondria are important regulators for SOCE, acting as the buffer of cytosolic Ca^2+^, thus limiting ER Ca^2+^ refilling and promoting SOCE [95,96]. The recent studies investigating the interplay between mitochondrial Ca^2+^ and SOCE in breast cancer cell migration established a potential link of SOCE to cancer cell metabolism. As the “power factory” of the cell, mitochondria play critical roles in cellular bioenergetics. Mitochondrial Ca^2+^ is believed to be a key regulator of mitochondrial oxidative phosphorylation, thereby contributing to the cellular energy production [97]. Abnormal regulation of mitochondrial Ca^2+^ is implicated in the reprogramming of cellular metabolism [98,99]. In human breast cancer MDA-MB-231 or Hs578t cells, silencing of mitochondrial Ca^2+^ uniporter (MCU), one of the primary transporters to mediate Ca^2+^ influx into mitochondria [100], impaired mitochondrial Ca^2+^ accumulation and attenuated SOCE, accompanied by impaired cell migratory ability [101,102]. Mechanistic investigations in Hs578t cells showed that that altered MCU expression regulated the activities of actin-regulating proteins Rho-GTPases and RhoA, as well as the Ca^2+^-dependent effectors Rac1 and Calpain [101]. However, the impact of STIM/Orai-mediated SOCE on cellular energy status, such as ATP productions or oxidative phosphorylation, was not examined in these cells.

## 5. Emerging Roles of SOCE Signals in the Tumor Microenvironment

The tumor microenvironment represents a dynamic interaction between cells, factors, and events that cover the effects on angiogenesis, tumor immunology, and cytokine/growth factor secretion to promote or suppress tumor progression [103,104]. The involvement and reliance on the SOCE dependent Ca^2+^ signal in the tumor microenvironment are still relatively unexplored.

### 5.1. Tumor Angiogenesis

Tumor angiogenesis, the recruitment of new blood vessels, is necessary to support the growth, expansion, and metastatic dissemination of developing tumors [105,106]. Vascular endothelial growth factor (VEGF)—a potent inducer of vascular endothelial cell proliferation and migration—is a critical regulator for the complex process of vascularization and tumor angiogenesis. The downstream signaling cascades of VEGF receptor involve the increase in the cytosolic Ca^2+^ levels via PLCγ activation [107,108].

The functional role for the STIM1-Orai1 dependent SOCE in vascular endothelial cells firstly came from a study showing that silencing of STIM1 or Orai1 in human umbilical vein endothelial cells (HUVECs) attenuated VEGF-induced Ca^2+^ influx and cell proliferation [109]. Interestingly, knockdown of STIM1, STIM2, or Orai1 inhibited the proliferation of HUVECs, but Orai1 knockdown showed a stronger effect as compared to STIM1 or STIM2 knockdown. A study further demonstrated the important role of Orai1-dependent SOCE in VEGF-activated human endothelial cell migration, in vitro tube formation, and in vivo angiogenesis in the chick chorioallantoic membrane [110]. The functional significance of STIM1/Orai1-dependent SOCE in tumor angiogenesis was confirmed by a study using the model of SiHa cervical cancer cells [30]. The secretion of VEGF of cervical cancer cells correlated with STIM1 expression levels, as demonstrated by overexpression or RNAi-mediated manipulation of STIM1 levels. In a mouse tumor xenograft model of cervical cancer, STIM1 silencing or SOCE blockade resulted in a reduction in tumor neovascularization, and tumor growth.

A recent study suggested a functional relevance of Orai3 in VEGF signaling and vascular endothelial cell remodeling [111]. Orai3 silencing in HUVECs reduced VEGF-induced tube formation in vitro and in vivo. Results from mechanistic investigations suggested that VEGF induces the production of arachidonic acid via the PLCγ1 pathway, and thus promotes the accumulation of Orai3 at the plasma membrane and Orai3-dependent Ca^2+^ entry.

Collectively, the contribution of augmented SOCE to tumor angiogenesis can be summarized as follows; (i) SOCE in cancer cells regulates the production of VEGF, which is critical for the formation of new blood vessels and (ii) SOCE in vascular endothelial cells regulates the proliferation and migration of these cells. Therefore, STIM1/Orai1-mediated Ca^2+^ machinery is a potential molecular target for strategies against tumor neovascularization.

### 5.2. Antitumor Immunity

Evasion of immune surveillance has been recognized as one of the new hallmarks of cancer [3]. The increased infiltration of immune cells into tumor microenvironment has long been regarded as the attempt of the immune system to eradicate tumors [2,3]. During tumor progression, cancer cells also acquire abilities to dictate the compositions and functions of immune infiltration to counteract effective antitumor immunity [112]. Some tumor cells are able to release immunosuppressive factors that induce the reprogramming of immune cells toward an immunosuppressive phenotype. For example, the immunomodulatory protein galectin-9 secreted by tumor cells are known to trigger apoptosis of mature helper T cells through stimulating their Tim-3 death-inducing receptor, allowing immune evasion [113,114].

The intracellular Ca^2+^ signal is required for modulating functions of innate and adaptive immune cells. SOCE deficiency due to STIM or Orai gene mutations can lead to functional defects of immune cells, including mast cells, macrophages, neutrophils, dendritic cells, natural killer cells, B cells, and T cells, and cause immunodeficiency in patients [115,116,117]. It is well established that STIM1/Orai1-mediated SOCE represents the predominant Ca^2+^ influx mechanism in lymphocytes [118,119], and is involved in many Ca^2+^-dependent T cell functions ranging from proliferation [120], immune synapse formation [121], to granule exocytosis [122]. Moreover, analyses of mice T cells with targeted deletion of STIM1 or STIM2 demonstrated that both STIM isoforms regulate SOCE of T cells, and such Ca^2+^ influx is essential for the development and function of regulatory T cells [123].

The essential role of SOCE mediated by STIM proteins for antitumor immunity was firstly emphasized by a study on the conditional knockout mice with T cell-specific disruption of both STIM isoforms, which exhibit a complete loss of SOCE in T cells [124]. Cytotoxic lymphocytes (CTLs) are key immune cells that play a crucial role in antitumor immune responses through their ability to kill tumor cells by releasing perforin or granzymes which cause caspase-dependent apoptosis in tumor cells [125], employing the Fas/FasL death receptor system [126], or secreting IFN-γ and TNF-α [127]. It was found that mice lacking STIM1 and STIM2 in CTLs fail to adequately control tumor cell engraftment and growth in vivo [124]. Mechanistically, STIM1 and STIM2 are crucial for the cytolytic effector functions of CTLs, especially the production of IFN-γ and TNF-α, the release of perforin-containing cytolytic granules, the induction of FasL, and thus tumor cell killing. Hence, SOCE in CTLs is required for efficient tumor-specific cytolytic functions, as well as for preventing engraftment of tumor cells in vivo. However, a recent study demonstrated that partial inhibition of Orai1 might paradoxically increase perforin-dependent cancer cell killing of CTLs [128], leading to a hypothesis that partial inhibition of Orai1-dependent SOCE may contribute to tumor elimination.

## 6. STIM1/Orai1-Dependent SOCE as Novel Targets for Cancer Therapy

As knowledge on the importance of SOCE in tumor biology and cancer progression accumulates, the blockade of STIM1/Orai1-dependent Ca^2+^ signaling has emerged as the potential and plausible targets for cancer therapeutics. Several small-molecule SOCE inhibitors have been developed, and some of which have been demonstrated with the potentials as a cancer therapy in preclinical animal studies. SKF-96365—one of the potent pharmacological blockers of SOCE—prevented tumor cell metastasis in a mouse model of breast cancer [82]. The blockade of SOCE by SKF-96365 also retarded the growth and angiogenesis of cervical cancer xenografts in mice [30]. Although SKF-96365 is not specific for STIM1/Orai1-mediated SOCE, further evidence from genetic approaches to overexpress or knockdown STIM1/Orai1 supported that SKF-96365 administration in these animals targeted the STIM1/Orai1 complex [30,82]. Similar to the effects of SKF-96365, another well-known small-molecule SOCE inhibitor 2-APB was reported to inhibit the growth and angiogenesis of cervical cancer xenografts in mice [30]. A recent study reported that Synta66, a relatively new potent and selective SOCE blocker developed by Synta Pharmaceuticals [129], impaired the migratory and proliferative capabilities of MDA-MB-468 triple-negative breast cancer cells in response to EGF stimulation [130]. Although not yet been examined in animal models, the results suggested that Synta66 or its structural analogs could be applied to further investigation for cancer therapeutics.

Until now, no SOCE inhibitors have been approved for the clinical use of cancer treatment [131,132,133,134], but few SOCE inhibitors have now reached clinical trials. Carboxyamidotriazole, a synthetic compound with nonselective, weak, indirect inhibition on SOCE [135,136], was reported to have antitumor and antiangiogenic properties in vitro and in vivo [137,138,139,140]. Carboxyamidotriazole has been investigated in preclinical studies and clinical trials in some newly diagnosed or relapsed solid tumors, including ovarian carcinoma, renal cell carcinoma, or glioblastoma [141,142,143]. A multicenter Phase IB trial for glioblastoma and anaplastic gliomas concluded that carboxyamidotriazole could be combined safely with chemoradiotherapy or the oral chemotherapy drug temozolomide and displayed promising central nervous system distribution and encouraging activity [143]. RP4010, a novel and potent SOCE blocker currently in Phase I/IB clinical development [144], was reported to have antitumor activities on B-cell lymphoma and esophageal cancer in vitro and in vivo [145,146]. RP4010 attenuates the tumor growth of diffuse large B-cell lymphoma as a single agent or as synergy in combination with known agents in a preclinical study [145]. These studies suggest that the modulation of SOCE could serve as maintenance therapy or extend the therapeutic efficacy for cancer treatment. A recent advance in the clinical development of small-molecule SOCE inhibitors came from the CalciMedica compound CM4620, which recently has completed Phase IIa clinical trials for treating moderate to severe acute pancreatitis [147]. The preclinical animal studies of acute pancreatitis demonstrated that CM4620 significantly ameliorated the severity and attenuated the features of inflammations in mouse and rat experimental models [148,149]. These compounds might hold promise for further investigations as therapy for certain types of cancer arising from aberrant overexpression of SOCE.

In recent years, investigations employing high-throughput screens have aided the development of novel SOCE modulators. For example, a cell-free protein interaction screening using minimal functional domains of STIM1 and Orai1 identified AnCoA4, which inhibited SOCE at sub-micromolar concentrations and blocked T cell activation in vitro and in vivo, from a small-molecule microarray of 12,000 compounds [150]. Such inhibition by AnCoA4 was due to the direct binding to the C-terminus of Orai1, inhibiting Ca^2+^ influx and also reducing binding to STIM1. A recent investigation used an in silico virtual screen to search for agents that share significant similarity in 3D shape and surface electrostatics with the best-known SOCE inhibitors from a library of >1800 FDA-approved drugs [151]. Results from this screening identified five novel compounds, i.e., leflunomide, teriflunomide, lansoprazole, tolvaptan, and roflumilast, with dose-dependent inhibition of SOCE in cell-based assays. Of these agents, leflunomide and its active metabolite, teriflunomide, significantly suppressed SOCE at clinically-relevant doses. Except for the small-molecule inhibitors of SOCE, a newly generated anti-Orai1 monoclonal antibody with specificity for the second extracellular loop was reported to cause cellular internalization of Orai1 and resulting in inhibiting in vitro T-cell activation and cytokine production and in vivo T-cell-mediated graft versus host disease in a mouse model [152]. Such results indicated the feasibility of antibody-mediated inhibition of Orai1 for the treatment of autoimmune diseases [152]. However, the potentials of the above mentioned newly identified SOCE inhibitors in cancer therapeutics remain to be clarified.

Despite the great potential of SOCE blockade in cancer treatment, the systemic use of SOCE inhibitors for cancer therapeutics should be cautious due to the ubiquitous expression of STIM and Orai protein, as well as their essential roles in the human immune system, including antitumor immunity. A potential solution is to develop SOCE modulators that specifically target tumor cells or tumor-associated vascular endothelial cells. To design assays in the presence of both cancer cells and immune cells will help the identification of the SOCE modulators for effective antitumor therapeutics. Another feasible strategy is to develop the therapeutic approaches targeting cancer cell-specific mechanisms of SOCE activation. For example, a study in the model of human cervical cancer indicated that the microtubule-associated histone deacetylase 6 (HDAC6) has different regulatory effects on the microtubule-dependent STIM1 translocation and SOCE activation between cancerous and noncancerous epithelial cells [24]. HDAC6—a unique cytosol-localized HDAC member—is known as a prominent α-tubulin deacetylase [153]. In cervical cancer cells, but not in noncancerous epithelial cells, hyperacetylation of α-tubulin by pharmacological blockade or silencing of HDAC6 abrogated microtubule-dependent STIM1 translocation and inhibit SOCE activation [24]. Therefore, HDAC6 can be a cancer-specific target of malignant phenotypes mediated by STIM1-dependent SOCE, at least for cancers with upregulation of HDAC6 and STIM1. Additionally, the plasma membrane localization of Orai proteins, one of the key steps leading to the full activation of SOCE, could be a potential therapeutic target. A recent study on breast cancer MCF-7 and MDA-MB-231 cells showed that the surface expression of Orai1 and Orai3, as well as the subsequent Ca^2+^ influx, was significantly reduced by overexpressing the dominant-negative mutant, or silencing, of the canonical transient receptor potential 6 (TRPC6) [154], implying the feasibility of targeting TRPC6 for cancer therapy.

## 7. Conclusions

**Figure 2:**
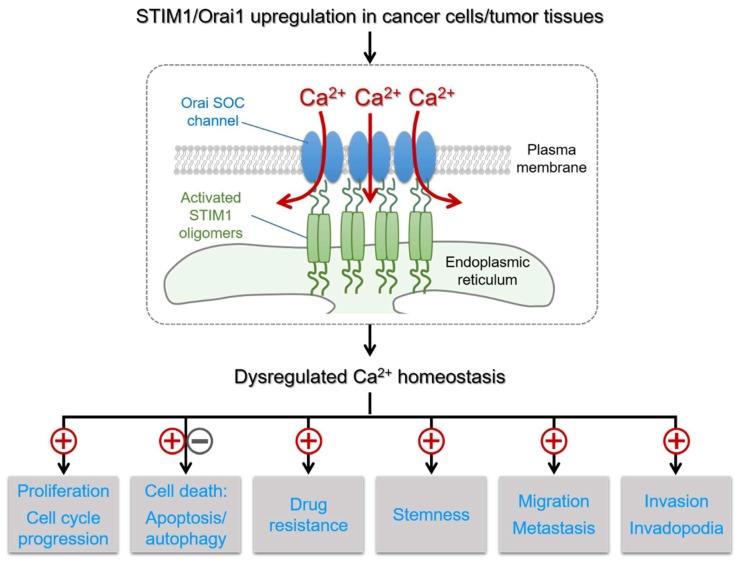
The active STIM1-Orai1 SOC channel complex and its important role in cancer. The effects of the STIM1/Orai1-mediated Ca^2+^ influx on several cancer hallmarks and cancer-related signaling pathways are indicated with ⊕ for promoting effects and ㊀ for inhibiting effects.

It has become clear that STIM/Orai-mediated SOCE plays an important role in a variety of malignant characteristics (Figure 2). Several studies have explored optimistic possibilities of targeting STIM1 and Orai1-mediated Ca^2+^ signaling in cancer cells, as well as vascular endothelial cells, for therapeutics. However, as some of the immune responses regulated by SOCE are cancer protective, a deeper understanding of the mechanisms and functions of SOCE in different types of immune cells in the tumor microenvironment is critical. Further studies aim at developing potent and selective inhibitors target cancer cell-specific SOCE pathways will facilitate better therapeutic approaches.

## Figures and Tables

**Table 1:** Summary of the diagnostic and prognostic values of STIM/Orai in human cancers.

SOCE Molecule	Cancer Type	Expression in Tumor		Diagnostic/Prognostic Significance	Reference
		mRNA	Protein		
STIM1	Cervical	N/A ^1^	↑	Tumor size: ↑ Lymph-node metastasis: ↑ Survival: ↓	[30]
STIM1	Colorectal	↑	↑	Poor differentiation Tumor invasion: ↑ Lymph-node metastasis: ↑	[32,33]
STIM1/ STIM2	Breast	↑	N/A	Survival: ↓	[45]
STIM2	Colorectal	↑	N/A	Cancer cell invasion: ↓	[43]
Orai1	Esophageal	N/A	↑	Overall survival: ↓ Recurrence-free survival: ↓	[40]
Orai1	Multiple myeloma	↑	↑	Progression-free survival: ↓	[37]
Orai3	Lung	N/A	↑	Higher tumor grades Visceral pleural invasion: ↑ Overall survival: ↓ Metastasis-free survival: ↓	[47,48]

^1^ N/A, not applicable.
